# Valve-Actuator-Integrated Reference Electrode for an Ultra-Long-Life Rumen pH Sensor

**DOI:** 10.3390/s20051249

**Published:** 2020-02-25

**Authors:** Shogo Higuchi, Hironao Okada, Seiichi Takamatsu, Toshihiro Itoh

**Affiliations:** 1 Department of Human and Engineered Environmental Studies, Graduate School of Frontier Sciences, The University of Tokyo, Kashiwa 277-8563, Japan; shiguchi@s.h.k.u-tokyo.ac.jp (S.H.); seiichi-takamatsu@edu.k.u-tokyo.ac.jp (S.T.); 2 Sensing System Research Center, National Institute of Advanced Industrial Science and Technology (AIST), Tsukuba 305-8564, Japan; hironao.okada@aist.go.jp

**Keywords:** pH sensor, reference electrode, liquid junction, shape memory alloy actuator

## Abstract

We demonstrated a newly developed Ag/AgCl reference electrode- with a valve-actuator for two years or longer rumen pH monitoring. Previous studies on pH sensors reported that the short lifetime of Ag/AgCl reference electrodes is caused by an outflow of internal electrolyte. We introduced a valve-actuator into a liquid junction to reduce the outflow by intermittent measurement. The results indicated that the potential change when switching the liquid junction was less than 0.5 mV and its response time was less than 0.083 s. In the 24-h potential measurement with the valve-actuator-integrated reference electrode (VAIRE), the valve was actuated once every hour, and the standard deviation of the potential was 0.29 mV. The lifetime of the VAIRE was estimated at 2.0 years calculating from an electrolyte outflow, which is significantly longer than that of conventional reference electrodes. A pH sensor using the VAIRE was estimated to operate for 2.0 years with the pH error ≤0.1, which meets the requirement of cows’ rumen pH monitoring.

## 1. Introduction

**Figure 1:**
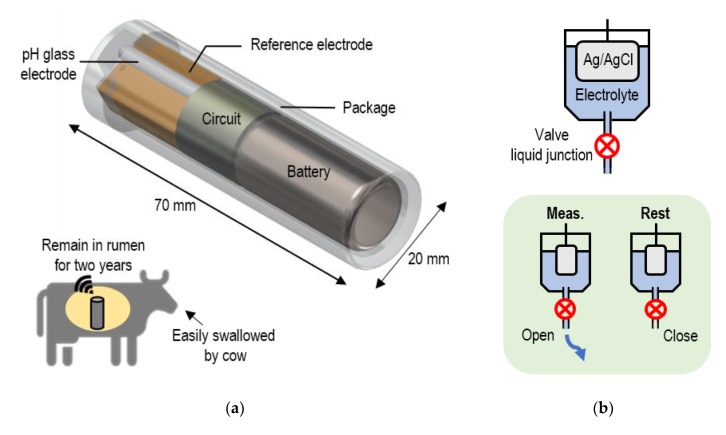
Schematic view of valve-actuator-integrated reference electrode (VAIRE) for cows’ rumen pH monitoring: (**a**) design of the wireless rumen pH sensor; (**b**) principle of the VAIRE.

In recent years, pH monitoring of a cow’s rumen has become increasingly important in the livestock industry because real-time pH monitoring can prevent a deadly disease called rumen acidosis. Rumen acidosis is caused by rapidly digestible carbohydrates for stable production of rich milk and marbled beef that contains a large amount of fat. When a cow suffers from rumen acidosis, the pH of the cow’s rumen decreases from approximately 7.0 to below 5.5. If the pH decrease can be detected at an early stage, administration of pH adjustment medicine will prevent serious condition [1]. Therefore, wireless pH sensors for cows’ rumen have been developed over the past ten years as a simple method of real-time pH monitoring [1,2,3,4,5]. We are also developing a rumen pH sensor shown in Figure 1. The size of the sensor is designed to be easily swallowed and remain in the rumen.

The lifetime of conventional pH sensors is at most three months due to the performance of a reference electrode. However, it is insufficient for use on a farm. It is desired to measure once every hour for two years with a pH error ≤0.1 without any maintenance because the feeding duration of beef cows is two years.

pH glass electrodes are the most established and commercially available pH working electrodes, but, in recent years, Ion Selective Field Effect Transistors (ISFETs) have been widely used. As long as the surface of the ISFET’s insulating film is not contaminated, the ISFET can be used without drift. Although surface contamination by rumen fluid is a concern, a pH drift of ±0.1 or less was achieved in a rumen measurement for 18 days by using an FET-based pH sensor with a protective layer in a previous study [5]. It should be noted that contamination is not fatal to the electrode.

Although considerable efforts have been made to develop new types of reference electrodes such as a reference electrode field effect transistor (REFET) [6,7] or solid-state Ag/AgCl reference electrodes [8,9,10,11,12], conventional Ag/AgCl reference electrodes have proven to be the most stable and accurate [6,13,14]. The potential is defined by an Equation (1) under the condition that the Equilibrium (2) holds:

(1) $$E=E^{0}-\frac{RT}{F}\ln\left(a_{{\mathrm{Cl}}^{-}}\right),$$

(2) $$\mathrm{AgCl}\left(s\right)+e^{-}↔\mathrm{Ag}\left(s\right)+{\mathrm{Cl}}^{-}\left(\mathrm{aq}\right)$$

where $E,E^{0},\;R,\;T,\;F,a_{{\mathrm{Cl}}^{-}}$ are the electrode potential, the standard electrode potential, the gas constant, the temperature, the Faraday constant, and the chloride ion concentration, respectively. The temperature in cow’s rumen is maintained between 38 °C and 41 °C, and the potential change due to the temperature fluctuation is 0.13 mV, which is negligibly small.

Ag/AgCl reference electrodes require routine maintenance to replenish their internal electrolyte. To reduce the outflow of electrolyte, a porous frit is routinely used as a liquid junction in commercial electrodes, but it often results in potential instability caused by clogging of AgCl complex from internal electrolyte and other contamination from analytes [15,16]. These results indicate that free-diffusion liquid junction reference electrodes are potentially more stable [14,17,18], but the flow rate of internal electrolytes is too large for the application to rumen pH sensors.

In this study, we developed a valve-actuator-integrated reference electrode (VAIRE) for an ultra-long-life pH sensor. Figure 1b shows the principle of the VAIRE. The lifetime can be extended by intermittent operation, in which the liquid junction is opened only during measurement [19]. We confirmed, for the first time, that the potential of the Ag/AgCl reference electrode was stable when switching the liquid junction, and we successfully fabricated the VAIRE. Its performance was evaluated by measuring impedance of the liquid junction, potential, and the pH value. We also estimated the lifetime by calculating an outflow of internal electrolyte.

## 2. Design

### 2.1. Potential Stability When Switching the Liquid Junction

The valve actuator is integrated into the Ag/AgCl reference electrode to reduce the electrolyte outflow by switching the liquid junction. The potential stability and response time when switching the liquid junction were investigated.

**Figure 2:**
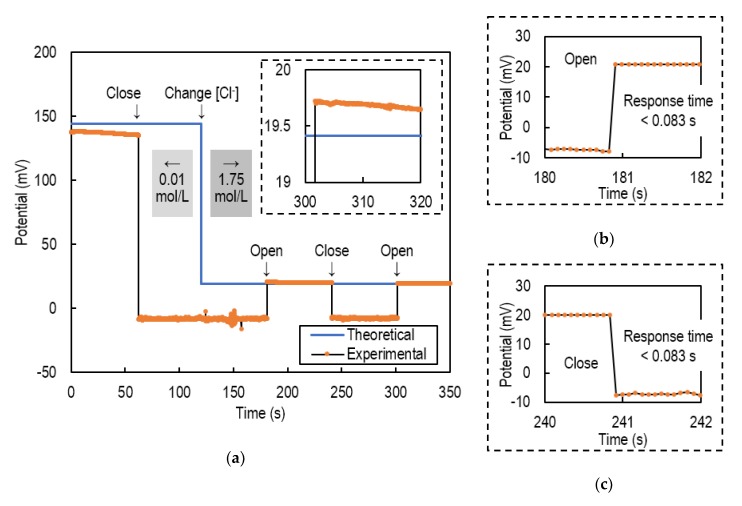
(**a**) Potential response of a Ag/AgCl reference electrode when its liquid junction was closed and then opened. The error from the theoretical value after switching was 0.5 mV or less; (**b**) enlarged view when opening; (**c**) enlarged view when closing.

Figure 2a shows the potential response of an Ag/AgCl reference electrode when its liquid junction was closed and then opened. In this experiment, the silicon tube was used as a liquid junction, and it was closed by manually pinching it with a clip. A bare Ag/AgCl electrode was used as a working electrode whose potential changed according to the chloride ion concentration in a test solution. At the start of the experiment, the concentration of the test solution was 0.01 mol/L and was changed to 1.75 mol/L by adding a saturated KCl solution during the experiment.

Even when the liquid junction was closed and then opened, a potential corresponding to the concentration was shown. The error from the theoretical value after switching was 0.5 mV or less. The response time was less than 0.083 s according to Figure 2b,c both at opening and at closing. The response time was less than 0.083 s even if the concentration of the KCl changed. Since this value is defined by the time resolution of the source meter, it should be noted that the actual response time may be smaller.

It was clarified that the potential change when switching the liquid junction was less than 0.5 mV. Moreover, the response time was sufficiently small for intermittent measurement such as 5-s measurement every hour.

### 2.2. Design of the Valve-Actuator-Integrated Reference Electrode

**Figure 3:**
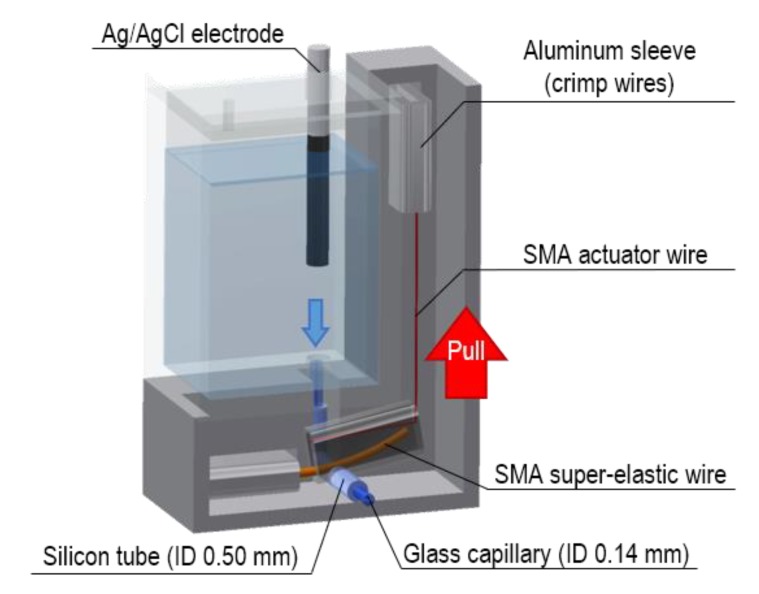
Design of the VAIRE using shape memory alloy (SMA) wires.

A design of the VAIRE using shape memory alloy (SMA) is shown in Figure 3. A bare Ag/AgCl electrode is immersed in a saturated KCl solution, and it flows out through a liquid junction. An SMA valve is attached to the liquid junction. Lead wires omitted in Figure 3 are connected to an SMA actuator wire.

This valve was designed as in the previous research [20]. While electric current is not flowing through the actuator wire, an SMA super-elastic wire holds the silicon tube, preventing the internal electrolyte from flowing to the outside. When voltage is applied and the actuator wire shrinks, the super-elastic wire is pulled, and then the tube opens accordingly. This valve is normally closed, which is suitable because of low power consumption for long-term monitoring application. Furthermore, SMA actuator has small volume and does not require high voltage (<3 V) [21].

### 2.3. Estimation of the Electrolyte Outflow

In this section, the amount of electrolyte to be stored inside the VAIRE is being calculated in order to have the lifetime of two years or more. In estimating the outflow, it is assumed that the valve is opened at 5 s every hour, and 20 kPa of differential pressure is applied inside the electrolyte container by injecting air before use.

The inner diameter of the pinched silicon tube was estimated from the impedance measurement of the liquid junction (Table 1). Since the electrolyte flow in the liquid junction is laminar flow, the flow rate can be calculated from the Hagen–Poiseuille equation:

(3) $$Q=\frac{\pi a^{4}∆p}{8\mu },$$

where Q is the flow rate, a is a dimeter of the tube, ∆p is differential pressure per unit length, and μ is viscosity. If the differential pressure is *P*, the flow rate of internal electrolyte is 3.5 *P* × 10^−3^ μL/h when opened and *P* × 10^−8^ μL/h when closed. The flow rate when closed is much smaller than when opened, so it will be treated as 0 μL/h in subsequent calculations.

**Figure 4:**
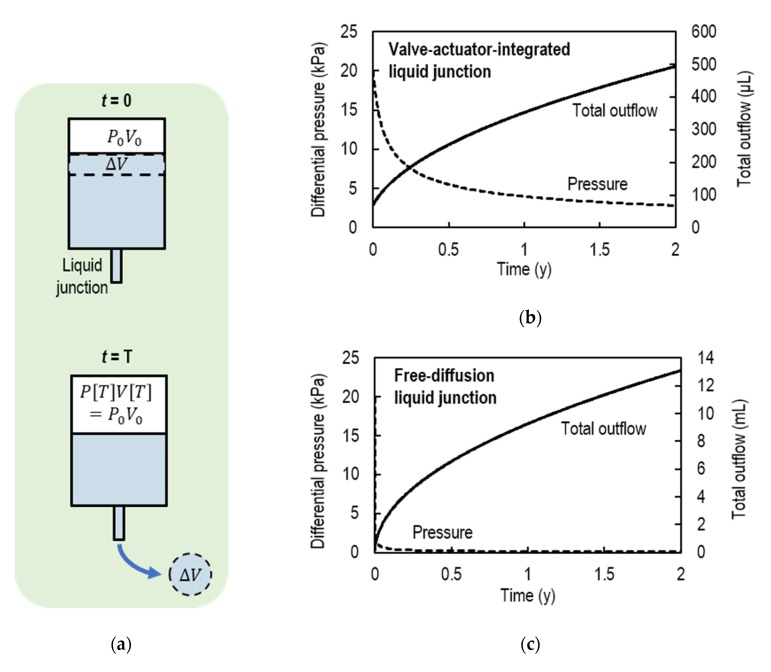
(**a**) Schematic view of assumptions when estimating the outflow over time from the flow rate; (**b**) estimation of the electrolyte outflow and differential pressure over two years when the VAIRE is used; (**c**) estimation of the electrolyte outflow and differential pressure over two years when the free-diffusion liquid junction reference electrode is used.

When estimating the outflow over time from the flow rate, two assumptions are made as shown in Figure 4a. First, the air injected into the container is an ideal gas in which Boyle’s law is satisfied as follows:

(4) P[t]V[t]=P[0]V[0],

where P[t] and V[t] is the pressure and the volume of the air in the container respectively. Second, the volume of electrolyte flowing out is equal to the volume of increasing air. This assumption can be represented as

(5) V[t]=V[0]+∫Qdt.

Equations (4) and (5) give an integral equation, and its solution is as follows:

(6) $$P\left[t\right]=\frac{V\left[0\right]}{\alpha }{\left(\frac{2P\left[0\right]V\left[0\right]}{\alpha }t+\frac{V{\left[0\right]}^{2}}{\alpha^{2}}\right)}^{-\frac{1}{2}},$$

(7) $$V\left[t\right]=\sqrt[{}]{2\alpha P\left[0\right]V\left[0\right]t+V{\left[0\right]}^{2}},$$

where α is the flow rate when the differential pressure is 1 Pa.

Figure 4b shows a graph of P[t] and V[t] when the VAIRE is used. V[0] is the electrolyte volume flowing out when the differential pressure of 20 kPa is applied for one hour with the valve opened. The pressure initially decreases significantly, and then gradually decreases. The total outflow was 5.0 × 10^2^ μL for two years. As a comparison, estimation when the free-diffusion liquid junction reference electrode is used is shown in Figure 4c. The total outflow was 13 mL for two years.

When the electrolyte volume of the VAIRE is 500 μL, the lifetime will be more than 2.0 years.

## 3. Materials and Methods

### 3.1. Fabrication of the Valve-Actuator-Integrated Reference Electrode

**Figure 5:**
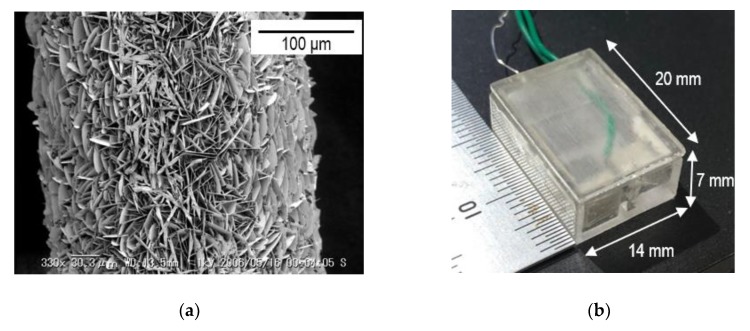
(**a**) Surface image of the bare Ag/AgCl electrode. Many sheets of AgCl on the silver wire were confirmed; (**b**) size of the completed VAIRE.

Bare Ag/AgCl electrodes were fabricated electrochemically. The procedure is as follows. First, a silver wire (diameter of 0.20 mm) was rinsed with acetone and then deionized (DI) water, followed by 4% HCl and then DI water again. Through this process, any organic and inorganic contamination on the silver wire was removed. Second, the silver wire as the anode and a platinum wire (diameter of 0.20 mm) as the cathode were immersed in a 1.0 M KCl solution. They were placed 10 mm apart, and both were dipped 10 mm into the solution. Finally, by applying a voltage of 3.0 V between two wires with a source meter (Keithley 2460-EC, Tektronix, Beaverton, OR, USA) for 30 min, AgCl was deposited on silver wire. The surface of the fabricated Ag/AgCl electrode is shown in Figure 5a. This image was taken with a scanning electron microscope (VE-8000, KEYENCE, Osaka, Japan). Many sheets of AgCl on the Ag wire were confirmed, which increases surface area and potential stability [22,23].

The SMA valve was fabricated with BMF150 (TOKI Corporation, Ota, Japan) as the SMA actuator wire, and MTL1-04 (ACTMENT, Kasukabe, Japan) as the SMA super-elastic wire. These two wires and lead wires were crimped using aluminum sleeves (Figure 3). The characteristics of SMA wires are summarized in Table 2. The liquid junction was made of silicon tube (SR1554, Tigers Polymer, Toyonaka, Japan) and glass capillary (2-453-01, AS ONE, Osaka, Japan). The container was fabricated using a 3D printer (Objet30Prime, Stratasys, Eden Prairie, MN, USA) and made of biocompatible plastic (Objet MED610, Stratasys, Eden Prairie, MN, USA). The container can store 500 μL of electrolyte from the estimation result in the previous section.

Figure 5b shows the size of the completed VAIRE. The volume of the free-diffusion liquid junction reference electrode whose lifetime is 2.0 years is 6.6 times larger than this VAIRE including the valve from the estimation result in the previous section.

### 3.2. Measurement

The impedance measurement of the liquid junction was conducted using an LCR meter (IM3533-01, HIOKI, Ueda, Japan) and two platinum wires with alternating current voltage of 10 mV and 10 kHz. The impedance change of the liquid junction by opening and closing the valve is detected with the LCR meter.

Electrode potential was measured with the source meter. A thermostatic chamber (FCI-280H, AS ONE, Osaka, Japan) was used not only to keep the temperature constant, but also to remove the effect of AgCl photosensitivity. The potential of the VAIRE was measured with respect to a free-diffusion liquid junction reference electrode whose potential drift could be regarded as zero.

In pH measurement, potential between a pH glass electrode (SP-GI-130, ASCH JAPAN, Hachioji, Japan) and the VAIRE was measured, and then the pH value was calculated.

## 4. Results and Discussion

### 4.1. Impedance of the Liquid Junction

**Figure 6:**
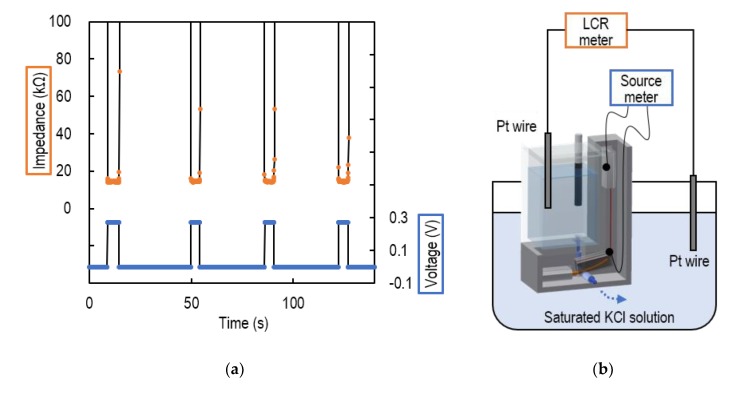
(**a**) Voltage applied to the SMA and measured impedance; (**b**) schematic view of a set-up of impedance measurement when the voltage was applied to the SMA of the VAIRE.

The operation of the valve was evaluated by measuring the liquid junction impedance when the voltage was applied to the SMA of the VAIRE. Figure 6a shows the applied voltage and measured impedance. Figure 6b shows the schematic view of a set-up of this experiment. The impedance decreased to about 15 kΩ when the voltage was applied to the SMA. Since the input impedance of the source meter used for the pH measurement is 10 GΩ, the impedance was negligibly small. When the voltage was reduced to zero volts, the impedance increased and became isolated. It was confirmed that the valve was switching between conduction and insulation of the liquid junction.

### 4.2. Intermittent Potential Measurement

**Figure 7:**
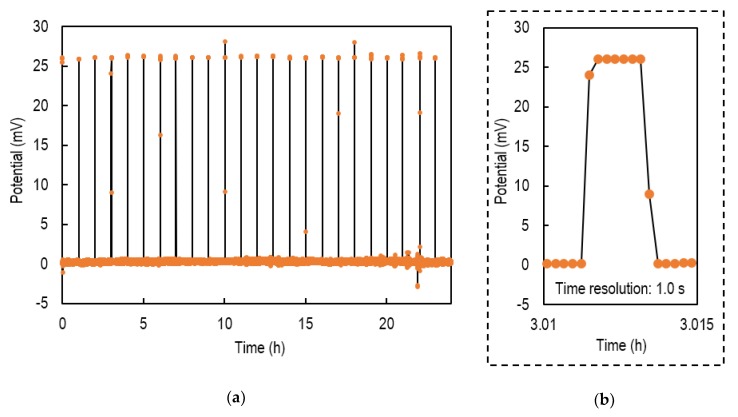
(**a**) Potential measurement of the VAIRE versus a free-diffusion liquid junction reference electrode; (**b**) enlarged view near 3.0 h. The maximum response time was 2.0 s.

Figure 7a shows the results of potential measurement using the VAIRE. In this experiment, the SMA was actuated at 5 s every hour.

During the measurement, the potential was about 26 mV once every hour, which indicated that the SMA valve switched between insulation and conduction for 24 h. The maximum response time was 2.0 s as shown in Figure 7b. The upper and lower outliers are considered to be noise during the valve opening and closing. The standard deviation of the potential, excluding outliers, was 0.29 mV. This value is expected to last as long as the electrolyte remains. Therefore, the standard deviation of the pH sensor applied the VAIRE was estimated at the pH of 0.005 for 2.0 years.

### 4.3. Iintermittent pH Measurement

**Figure 8:**
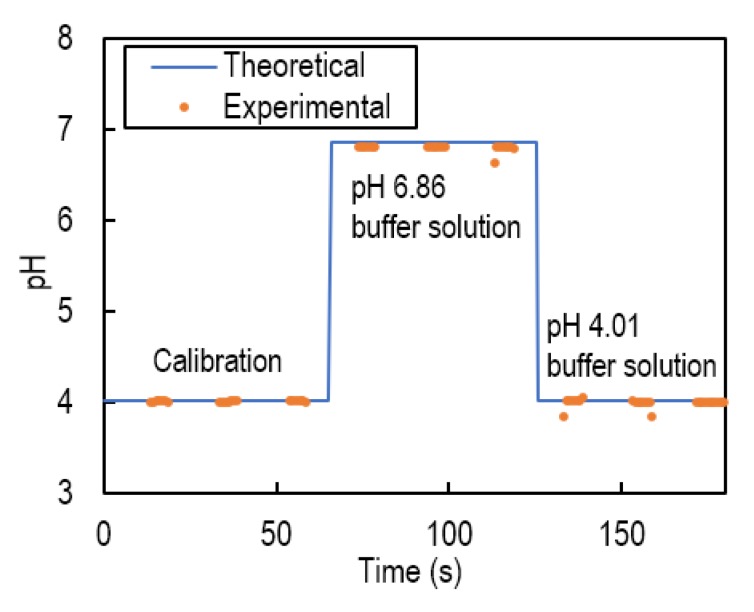
Intermittent pH measurement with the VAIRE and the pH glass electrode when changing the pH value of the test solution.

Figure 8 shows the results of intermittent pH measurement using the VAIRE and a pH glass electrode. The potential was measured while applying voltage to the SMA.

The potential varied according to a pH change of the test solution. After calibration was performed using a pH 4.01 buffer solution, the measured value of the pH 6.86 buffer solution was 6.79, so the error was 0.07, which satisfied the requirement of rumen pH monitoring.

## 5. Conclusions

The VAIRE was developed for ultra-long-life rumen pH sensors. By intermittent pH measurement, the VAIRE can reduce the electrolyte outflow. It was experimentally clarified that the potential change when switching the liquid junction of Ag/AgCl reference electrodes was less than 0.5 mV and the response time was sufficiently small for intermittent measurement such as 5-s measurement every hour. According to the estimation of the electrolyte outflow, the lifetime of VAIRE storing 500 μL of electrolyte is more than 2.0 years. The VAIRE was designed and fabricated using the SMA actuator, and its size is 14 mm × 20 mm × 7 mm. As for the fabricated VAIRE, it was confirmed by impedance measurement that the valve was switching between conduction and insulation of the liquid junction. In the 24-h potential measurement, the valve was actuated once every hour, and the standard deviation of the potential was 0.29 mV. A pH sensor using the VAIRE was estimated to operate for longer than 2.0 years with the pH error ≤0.1, which satisfied the requirement of rumen monitoring sensor.

## Figures and Tables

**Table 1:** Estimation of inner diameter of the pinched silicon tube.

	Open	Close
(A) Impedance of the liquid junction	17 kΩ	1 × 10^5^ kΩ
(B) Impedance of the glass capillary ^1^	8.6 kΩ	8.6 kΩ
Impedance of the pinched silicon tube ((A)–(B))	7.3 kΩ	1 × 10^5^ kΩ
Inner diameter of the pinched silicon tube	6.8 × 10^−2^ mm	6 × 10^−4^ mm

^1^ Calculated from inner diameter, length and conductivity of a KCl solution.

**Table 2:** Characteristics of the SMA actuator wire and the SMA super-elastic wire.

	SMA Actuator Wire	SMA Super-Elastic Wire
Material	Ni-Ti	Ni-Ti
Diameter	0.15 mm	0.40 mm
Length	10 mm	10 mm
Transformation temperature	70 °C	5 °C
Power	54 mW	-
